# Psychometric characterization of incidental feedback sources during grasping with a hand prosthesis

**DOI:** 10.1186/s12984-019-0622-9

**Published:** 2019-12-10

**Authors:** Meike Annika Wilke, Christian Niethammer, Britta Meyer, Dario Farina, Strahinja Dosen

**Affiliations:** 1 Department of Biotechnology, University for Applied Sciences Hamburg, Hamburg, Germany; 2 Advanced Rehabilitation Technology (ART) Lab, Department for Trauma Surgery, Orthopaedics and Plastic Surgery, Universitätsmedizin Göttingen (UMG), Göttingen, Germany; 3 Department of Computer Science, Eberhard-Karls University Tübingen, Tübingen, Germany; 4 Department of Bioengineering, Imperial College London, London, UK; 5 Department of Health Science and Technology, Center for Sensory-Motor Interaction, Aalborg University, Aalborg, Denmark

**Keywords:** Myoelectric prostheses, Upper limb, Artificial sensory feedback, Incidental feedback, Vibrotactile stimulation

## Abstract

**Background:**

A prosthetic system should ideally reinstate the bidirectional communication between the user’s brain and its end effector by restoring both motor and sensory functions lost after an amputation. However, current commercial prostheses generally do not incorporate somatosensory feedback. Even without explicit feedback, grasping using a prosthesis partly relies on sensory information. Indeed, the prosthesis operation is characterized by visual and sound cues that could be exploited by the user to estimate the prosthesis state. However, the quality of this incidental feedback has not been objectively evaluated.

**Methods:**

In this study, the psychometric properties of the auditory and visual feedback of prosthesis motion were assessed and compared to that of a vibro-tactile interface. Twelve able-bodied subjects passively observed prosthesis closing and grasping an object, and they were asked to discriminate (experiment I) or estimate (experiment II) the closing velocity of the prosthesis using visual (*VIS*), acoustic (*SND*), or combined (*VIS + SND*) feedback. In experiment II, the subjects performed the task also with a vibrotactile stimulus (*VIB*) delivered using a single tactor. The outcome measures for the discrimination and estimation experiments were just noticeable difference (JND) and median absolute estimation error (MAE), respectively.

**Results:**

The results demonstrated that the incidental sources provided a remarkably good discrimination and estimation of the closing velocity, significantly outperforming the vibrotactile feedback. Using incidental sources, the subjects could discriminate almost the minimum possible increment/decrement in velocity that could be commanded to the prosthesis (median JND < 2% for *SND* and *VIS + SND*). Similarly, the median MAE in estimating the prosthesis velocity randomly commanded from the full working range was also low, i.e., approximately 5% in *SND* and *VIS + SND*.

**Conclusions:**

Since the closing velocity is proportional to grasping force in state-of-the-art myoelectric prostheses, the results of the present study imply that the incidental feedback, when available, could be usefully exploited for grasping force control. Therefore, the impact of incidental feedback needs to be considered when designing a feedback interface in prosthetics, especially since the quality of estimation using supplemental sources (e.g., vibration) can be worse compared to that of the intrinsic cues.

## Background

Humans can effortlessly grasp and manipulate objects of very different properties, from heavy and robust to delicate and fragile. This is possible thanks to a sophisticated musculoskeletal structure innervated by a network of sensorimotor nerves, providing advanced motor commands and a comprehensive multimodal feedback (e.g., touch, proprioception, force). The studies in human motor control demonstrate that the somatosensory feedback is indeed instrumental for the planning and execution of grasping [1–3].

After an amputation of the hand, the motor and sensory functions are lost. The lost motor functions can be restored to a certain degree using myoelectric prostheses. These systems are controlled by recording the electrical activity of the user’s muscles to estimate the motion intention, which is then translated into prosthesis commands [4]. Typically, the wrist and hand flexor and extensor muscles are used to command prosthesis closing and opening proportionally, thereby resulting in an intuitive connection between the user’s brain and the artificial device [5]. However, current prosthetic systems are controlled in open loop, without explicitly providing any somatosensory feedback on the prosthesis state (e.g., hand aperture or grasping force) to the user. In order to truly compensate for the missing biological limb, it is commonly assumed that a prosthetic system needs to establish a bilateral communication to the user’s brain, by restoring both motor and sensory functions [6, 7].

Intuitive methods to provide somatosensory feedback have been a research topic for several decades [8], and substantial progress was made in recent years [9–12]. However, a commercially available solution capable of improving the prosthesis performance in the activities of daily living is still unavailable [13, 14]. There is a single commercial prosthesis (VINCENTevolution 2, Vincent Systems, DE) equipped with a simple vibratory feedback on the grasping force, but its clinical and functional utility has not been yet demonstrated. From the technical viewpoint, the non-invasive artificial feedback can be implemented using relatively simple solutions. A common approach is to use sensory substitution, where the lost sensory information is transmitted using alternative sensory modalities that are still spared following the amputation [15]. To this aim, the prosthesis is equipped with force and position sensors, and the sensor data are transmitted to the user by stimulating the skin of the residual limb to activate the tactile sense. The stimulation can be delivered using low-intensity electrical pulses [16] or vibration motors [17], and the information is conveyed by modulating the stimulation parameters. For example, vibration intensity and/or frequency can be proportionally associated to hand aperture [18] or grasping force [18–21]. In addition, the feedback can be provided using direct mechanical stimulation through skin stretch [22], movement on the skin [23], pressure cuffs/braces [24], or linear pushers [25–27]. More recently, several invasive solutions that directly stimulate the peripheral nerves [28–33] or somatosensory areas of the brain [34] have been presented.

Despite several methods have been successfully implemented and tested for restoring sensory feedback, the actual benefits of the artificial feedback are still elusive. Some studies showed significant improvements in performance with artificial somatosensory feedback [35–37]. However, these studies were conducted while controlling a virtual setup [35–37] and/or while blocking the incidental (visual and auditory) feedback sources [18, 31, 36, 38]. Some recent studies showed benefits of feedback in realistic, clinical settings [32, 39–43]. However, other experiments in realistic settings failed to show functional improvements in performance [13, 19, 20, 44, 45] or demonstrated some benefits of feedback only in specific conditions [13, 14, 40].

A major confounding issue when studying the effect of artificial feedback is the existence of incidental sensory information that is already present in prosthesis control. The user can observe the prosthesis motion (visual feedback) and hear the motor sound, which together with proprioception has recently been shown to already allow for a rather good control of grasping force [46]. In addition, motor vibrations as well as mechanical interaction with the object can propagate through the socket to be felt by the user. The user can exploit these incidental information sources to close the control loop, even in the absence of an explicit somatosensory feedback. For example, in a typical myoelectric prosthesis, the velocity of closing is related to the resulting grasping force, i.e., the faster the hand closes the stronger the force produced after contact (see Fig. 2 in [47]). Therefore, the information on the closing velocity can be employed by the prosthesis user to improve the control of grasping force, as demonstrated in [48]. Importantly, the closing speed can be estimated through visual observation or by listening to the motor sound.

Although it has been demonstrated that human subjects can indeed use such information for prosthesis control, the psychometric properties of the incidental sources of feedback have not been investigated yet, in contrast to psychometric tests of the artificial feedback methods (electro- and vibrotactile stimulation) that are thoroughly documented in the literature [16, 17].

In the present study, we systematically investigated the quality of the incidental prosthetic feedback both objectively and subjectively. To this aim, the psychometric properties of the incidental auditory and visual feedback produced by prosthesis motion were quantified in a discrimination task (Experiment I) and then compared to that of a simple vibration interface in an estimation task (Experiment II). The task for the subjects was to estimate the velocity of prosthesis closing using incidental feedback. Vibratory feedback was selected for comparison as a common method to provide explicit somatosensory feedback in prosthetics. The results demonstrated that the subjects could interpret the incidental information sources rather well, and even better than the supplemental vibrotactile feedback. These observations provide insights on possible reasons for the inconsistent results related to the benefits of sensory feedback in prosthetics and thereby guide the design of novel, more effective feedback solutions.

## Methods

### Participants

Twelve able-bodied volunteers (24 ± 3 years old, between 18 and 27 years, 6 women, 6 men) participated in the two experiments. The subjects were informed about the experiments and confirmed that they understood the experimental procedure. They gave informed consent to take part in the study and were reimbursed with 10 Euro per hour of participation. The study was approved by the ethics committee of the Universitätsmedizin Göttingen. Ten of the participants were right handed, two left handed. Four had corrected vision. All subjects had taken part in one previous study on prosthetics [46] but were otherwise naïve to prosthesis control.

### Experimental setup

**Fig. 1:**
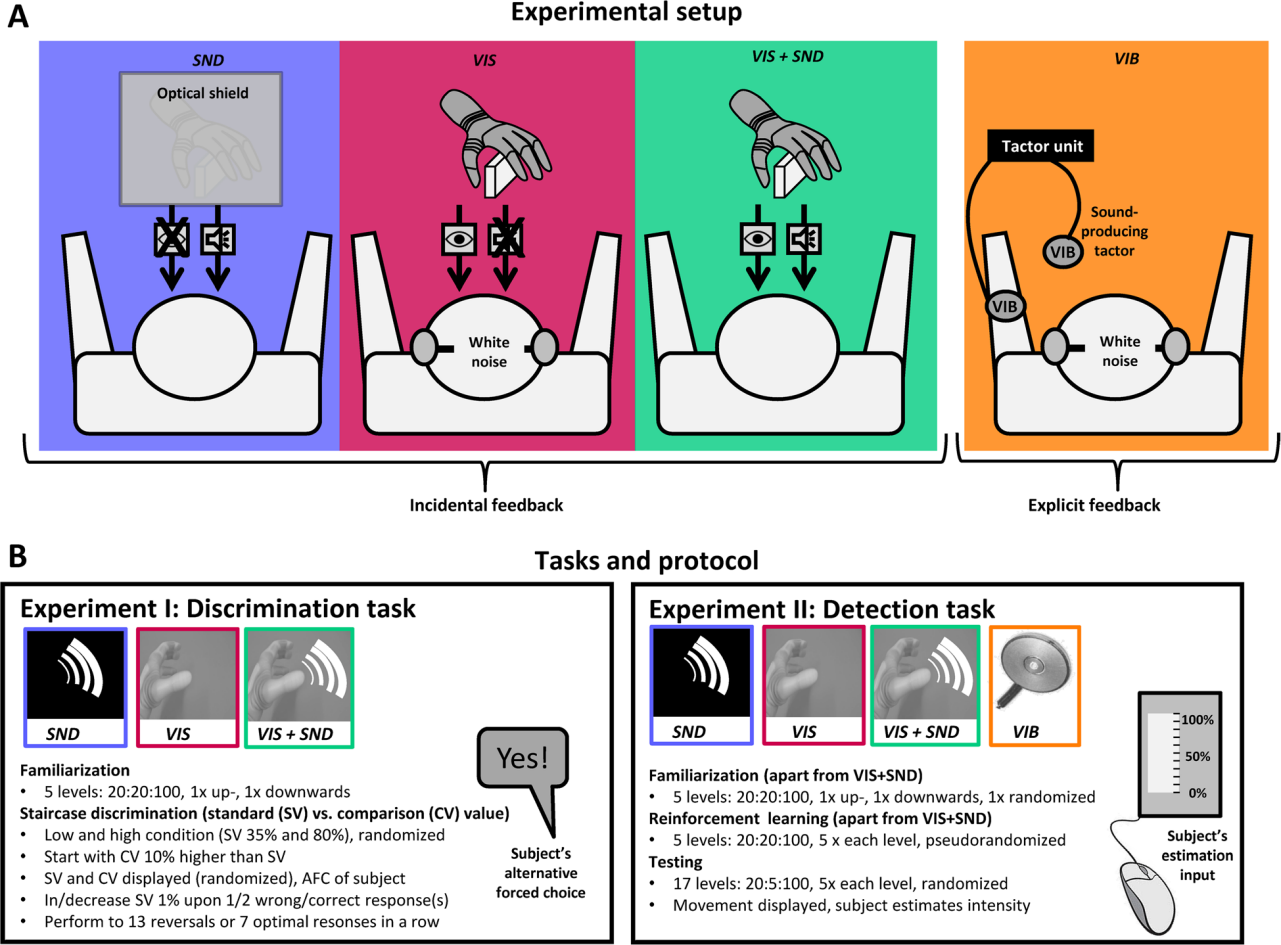
Experimental setup and protocol. **a** The prosthesis was commanded to close at different velocities (grasping forces) while the subjects perceived incidental sound (*SND*, blue), visual (*VIS*, red) or combined visual and sound (*VIS + SND*, green) feedback or vibration feedback (*VIB*, orange, in Exp II only). For the three incidental feedback conditions (*SND, VIS, VIS + SND*), the setup included a prosthetic hand grasping a wooden block. For *SND*, the subjects were prevented from seeing the prosthesis by a screen; during *VIS,* earphones played white noise to block the sound. For *VIB,* the setup included two C2 tactors, one strapped to the subjects’ left arm displaying the vibration that had to be interpreted, the other hanging freely in the air, producing a strong sound to block the sound of the first tactor, together with white-noise playing headphones. **b** For each experiment (I and II) and each feedback condition (except *VIS + SND*), there was a familiarization, reinforcement-learning, and testing phase. CV: Comparison value, SV: Standard value. 20:5:100 stands for 5% steps from 20 to 100%. See text for details

The current study was divided into two experiments, which were performed on different days, with Experiment I (Exp I) being at least 2 weeks before Experiment II (Exp II). The setup was similar in both experiments, as shown in Fig. 1a. It comprised 1) a prosthetic hand (Michelangelo prosthetic hand, Otto Bock Healthcare GmbH, Vienna, AT), controlled via Bluetooth, 2) a standard desktop PC with a 22″ computer screen (hidden from the subject’s view) controlling the prosthesis, 3) headphones playing white noise, and 4) a wooden block. In Exp II, the setup was extended by 5) two C2 tactors with a control unit (Engineering Acoustics, US) connected to the PC via a USB port to deliver vibrations, and 6) a laptop with a mouse used by the subject to indicate answers with the right hand (as explained below). The subjects were seated in a chair with the prosthetic hand in a table-top position in front of them at a distance of approx. 0.5 m, allowing a lateral view on the prosthesis (thumb-side, see Fig. 1). When closed using pinch grip, the prosthesis grasped a wooden block, which was fixed to the table. In Exp II, one C2 tactor was placed on the ventral aspect of the forearm of the subject’s left arm. The subject placed the arm comfortably on a sideboard. The second vibrator was hanging freely in the air and was activated at the maximum intensity in each trial with vibration feedback, effectively preventing the subject to use the sound produced by the first vibrator as potential source of feedback. In addition, the subject wore headphones playing white noise.

The prosthesis and the tactors were controlled by a control-loop implemented in Matlab Simulink 2015b (Mathworks, US) using the prosthetic closed-loop test bench [49] that operated in real-time at 100 Hz. In this study, the prosthesis was controlled by a computer, i.e. the subjects did not control the prosthesis themselves, making them passive observers. During a single trial, the prosthesis closed with constant velocity, simulating a routine-grasping protocol [46]. Across trials, the closing velocity was changed according to the psychometric test procedure (explained below). In Exp I, the subjects verbally reported their discrete answers to the experimenter, whereas in Exp II they used the mouse to indicate the answer via a Matlab GUI.

### Experiment I: discrimination task

The aim of the first experiment was to assess the just noticeable difference (JND) in the incidental feedback using a staircase procedure. The JND is the minimal change in the amplitude of the feedback variable that can be perceived by the subject [50], therefore determining the effective resolution of the feedback channel. In the context of the present study, the JND corresponds to the minimal change in the prosthesis closing velocity that can be perceived by looking at the prosthesis (visual JND) and/or by listening to the prosthesis sound (auditory JND).

The experiment started with a familiarization phase in which the subjects watched as the prosthesis closed 10 times. The closing velocities changed from 20 to 100% of the maximum velocity (in steps of 20%), which corresponds to absolute average closing speeds between 38 mm/s and 324 mm/s (see Fig. 8 in the Appendix). This sequence was repeated twice. The subjects could see and hear the prosthesis while it closed.

**Fig. 2:**
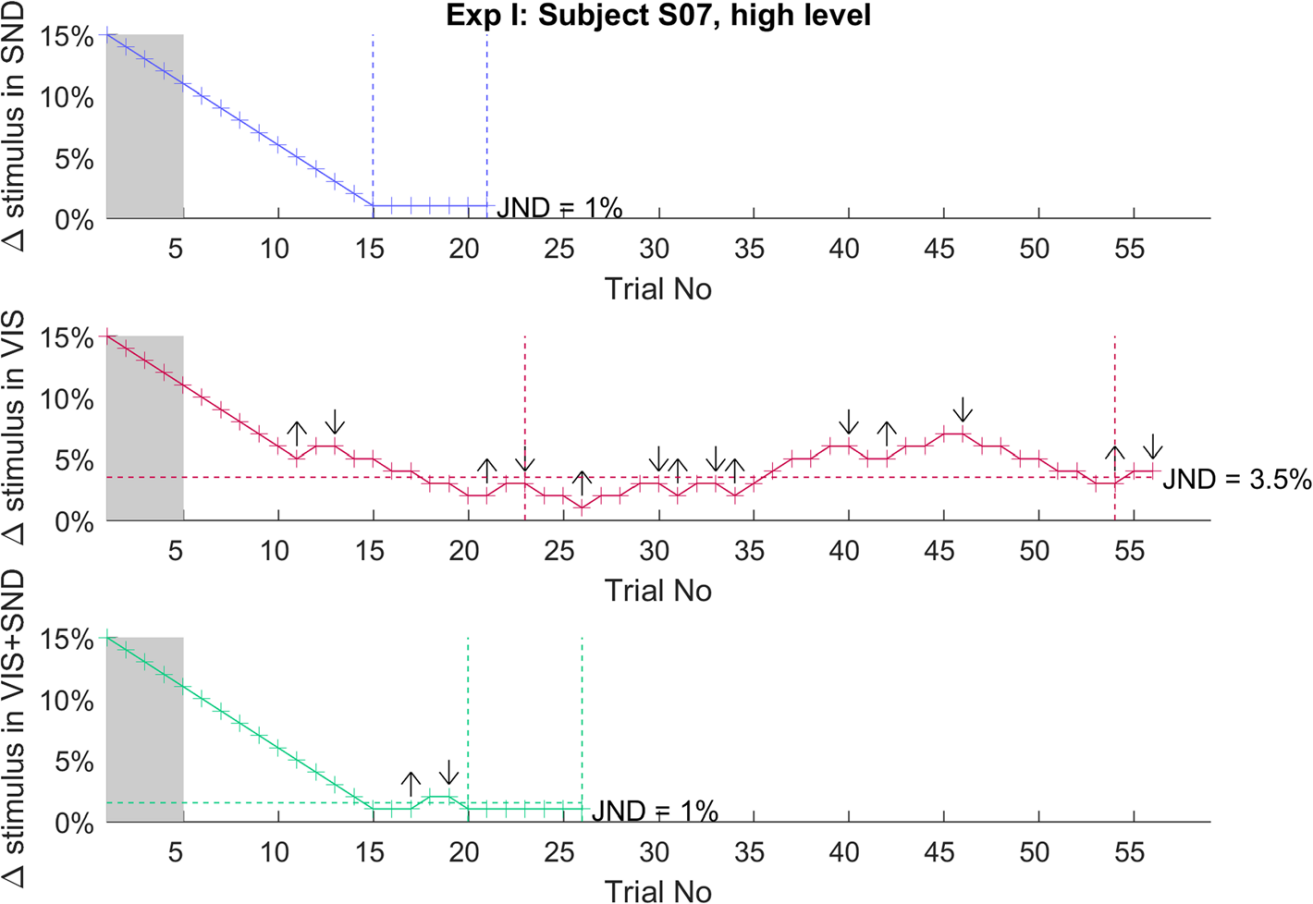
A staircase procedure for one representative subject in Exp I. The time course of the difference (Δ) between comparison and standard value recorded in Exp I across trials of a staircase procedure for Subject 7 at the high standard-value level (80%) for the three feedback conditions *SND* (top, blue), *VIS* (middle, red), and *VIS + SND* (bottom, green). The first 5 trials (grey underlay) reflect the reinforcement-learning phase. After that, wrong answers led to a 1% increase of the comparison stimulus, while two correct answers were needed for a 1% decrease (1 up/2 down staircase). The 10 reversals (indicated by an up-downward arrow) in the area between the vertical dashed lines (4th to 13th reversal) were used to calculate the JND, which is indicated by a horizontal dashed line. If the subject’s response was correct 7 times in a row at a 1% difference between comparison and standard stimulus, the JND was set to 1% and the run was terminated (see *SND* and *VIS + SND*)

The JND was assessed using a transformed 1 up / 2 down staircase procedure in a two-alternative forced-choice (= 2AFC) task, which targets 70.71% correct performance [50]. Each step of the staircase procedure comprised two trials of prosthesis closing. The prosthesis closed once at a fixed predefined velocity (standard value) and once at a velocity (comparison value) that was changed adaptively across trials based on the participant’s previous answers. In each trial, the standard and comparison value were provided in random order. The prosthesis stayed for 1 s in the opened or closed position, before closing or opening again. The comparison value was always higher than the standard value. The subjects observed the prosthesis using the provided auditory information (*SND*), visual information (*VIS*), and visual and auditory information (*VIS + SND*), depending on the condition, and they were then asked to report in which trial the prosthesis closed faster. If subjects answered correctly two times in a row, the comparison value was reduced by 1%. If they made a mistake, it was increased by 1%. The first five steps of the staircase were used for reinforced learning, where the experimenter revealed the correct response, to make sure that the subjects understood the task. The initial comparison velocity was set to be 15% higher than the standard one, which was a large difference and hence simple to discriminate. After the reinforcement learning, the normal staircase procedure resumed, i.e., the subjects received no more feedback on the correct answer. The staircase procedure was terminated if 13 reversals occurred, with a reversal being the change of the comparison value from increasing to decreasing or vice versa (see Fig. 2, *VIS* for an example). The JND (staircase outcome) was then calculated as the difference between the mean of the last 10 reversals and the standard value. The procedure was also terminated if the comparison value was only 1% higher (i.e., as low as possible) than the standard value 7 times in a row (see Fig. 2, *SND* and *VIS + SND* for an example). In this case, the JND was set to 1%. To assess the JND at both low and high velocities, the staircase was performed two times, at the standard values of 35 and 80%, respectively (order pseudorandomized across subjects), that correspond to absolute average closing speeds of 72 mm/s and 231 mm/s (see Fig. 8 in the Appendix).

The JND was measured in several conditions with different implicit feedback. In *SND* the vision was blocked by a screen in front of the prosthesis, which made only the closing sound available. In *VIS*, the subjects could see but not hear the prosthesis since the sound was blocked by noise-cancelling earphones playing white noise. In the final condition (*VIS + SND*), the subjects could normally hear and see the prosthesis. The order of the feedback conditions was pseudorandomized across subjects. Generally, the subjects were encouraged to focus on every detail of the closing and grasping process that they deemed could be useful to evaluate the presented closing velocity. This included the interaction between the wooden block and the prosthesis, the duration of the closing process, the frequency and intensity of the sound, etc. All these possibilities were explained to the subject beforehand.

The JND was computed for each subject, feedback condition, and standard-value velocity. As Kolmogorov-Smirnov tests showed that the data were not normally distributed, non-parametric tests were used and medians and interquartile ranges are reported in the manuscript. To compare the three feedback conditions, the subjects’ JNDs were averaged across the two standard stimuli and a Friedman test was applied. Upon significance, the conditions were compared pairwise using three Bonferroni-corrected Wilcoxon signed-rank tests. For comparison between low and high velocities separately for each feedback condition, three Bonferroni-corrected Wilcoxon signed-rank tests were performed. The threshold for statistical significance was set to *p* < 0.05.

### Experiment II: estimation task

The aim of the second experiment was to assess how well the subjects could estimate the prosthesis closing velocity using different sources of incidental feedback. In this case, the prosthesis closed at a constant but arbitrary velocity and the subjects were asked to provide an estimate of the closing speed (absolute estimation versus relative discrimination in Exp I). In addition, the subjects’ ability of estimating the intensity/frequency of vibrotactile stimulation was assessed, a method which is commonly used in prosthetics to implement sensory feedback [10].

The experiment comprised four feedback conditions, three with implicit (*SND*, *VIS*, and *VIS + SND)*, and one with vibrotactile feedback (*VIB*). Each condition comprised a familiarization phase, a reinforcement-learning phase, and a testing phase. The only exception was *VIS + SND*, where only the test phase was implemented to assess the potential fusion of information from the incidental feedback sources.

During the familiarization phase in *SND* and *VIS*, the prosthetic hand was closed at five velocities (20, 40, 60, 80, and 100%) first in increasing, then in decreasing, and finally in random order. In the reinforcement-learning phase, the prosthesis closed randomly five times at each of these velocities (25 trials). After each trial, the subjects were asked to estimate the closing speed and the experimenter then informed them about the correct response. In the testing phase (correct answer not provided), the prosthesis closed at 17 velocities (20–100% with 5% steps), five times per velocity in random order. Importantly, the subjects were not informed about the discrete step-sizes (5%) but were told that any velocity between 20 and 100% might occur, in order to eliminate a bias towards the 5% steps. After the prosthetic hand closed, the subjects reported their closing-speed estimate by a mouse click to an analog scale (20–100%) shown at the laptop.

For the vibrotactile condition, the protocol was similar except that the task was to estimate the intensity/frequency of a vibrotactile stimulus (the prosthesis was not used). In each trial, a 2.5 s vibration was delivered using a C2 tactor fixed on the subjects’ left arm. The same levels and number of trials were used as in *VIS* and *SND.* The levels were implemented by simultaneously changing the frequency and intensity of stimulation. The dual-parameter modulation was used to facilitate the discrimination. The frequency (1–100%) ranged from 30 Hz to 270 Hz, and the intensity (1–100%) was chosen between 8 and 71% of the maximum intensity. Therefore, this condition was equivalent to using vibration feedback to transmit the prosthesis closing velocity via linear mapping.

The order of the four feedback conditions was randomized between subjects. However, the *VIS + SND* was always placed after *VIS* and *SND* such that the subjects received the same amount of learning in each of the two modalities before the fusion of modalities (*VIS + SND*) was tested. To investigate to which extent specific features were important when interpreting the feedback in each condition, the subjects were asked to indicate how much they relied on each feature using a visual-analog scale (0–10) in a questionnaire. They indicated how much they had focused on the speed of closing, duration of closing, and mechanical interaction between the prosthesis and the object for *VIS*, duration, frequency, and loudness of sound for *SND*, and frequency and intensity of vibration for *VIB*. In addition, in each condition they were asked to estimate their average absolute deviation from the correct value, to assess how confident they were in their estimation.

For evaluation, the success rate for the reinforcement-learning phase and the estimation errors in the testing phase were calculated for each trial, separately for each subject, level, and feedback condition. Again, non-parametric statistics were used. For the analysis of the reinforcement learning, the subjects’ success rate, averaged across the five tested levels, was compared across the three trained feedback conditions using a Friedman test and, upon significance, three Bonferroni-corrected Wilcoxon signed-rank tests were applied. In the testing phase, the median of the estimated value was computed for each level and feedback condition, reflecting whether and how much the subject over- or underestimated the correct level. For each level (except for the lowest and highest where over- and undershooting were not possible, respectively) and feedback condition, Bonferroni corrected Wilcoxon signed-rank tests were used to assess whether the subjects consistently over- or underestimated the level, by subtracting the estimated from the correct value and testing whether that value differed significantly from zero. Then, by pooling the absolute errors of all respective trials, the median absolute error (MAE) was determined for each subject and feedback condition, independently of the level. To test for differences between feedback conditions, a Friedman test and, upon significance, six Bonferroni-corrected Wilcoxon signed-rank tests on the MAE were used.

For analysis of the subjective results from the questionnaire, for both *SND* and *VIS* as well as separately for the acoustic and the visual features in *VIS + SND,* a Friedman test was applied and, upon significance, three Bonferroni-corrected Wilcoxon signed-rank tests were used for pairwise comparison. For *VIB*, a single Wilcoxon signed-rank test was used (only two features available). Six Bonferroni-corrected Wilcoxon signed-rank tests compared whether the feature usage differed when using one-feedback modality (*SND or VIS*) instead of full feedback (*VIS + SND*), e.g., the subjective reliance on the auditory duration of the prosthesis closing was compared between *SND* and *VIS + SND* condition. In addition, a dominance towards visual versus auditory features was explored by comparing the amount of reliance on the visual versus auditory features in *VIS + SND*, indicating which modality captured more attention when the full implicit feedback was available. To that end, in the *VIS + SND* condition for each subject the most strongly used auditory and visual features were selected across the three possible features per modality and these two were compared via a Wilcoxon signed-rank test.

## Results

### Experiment I: discrimination task

**Fig. 3:**
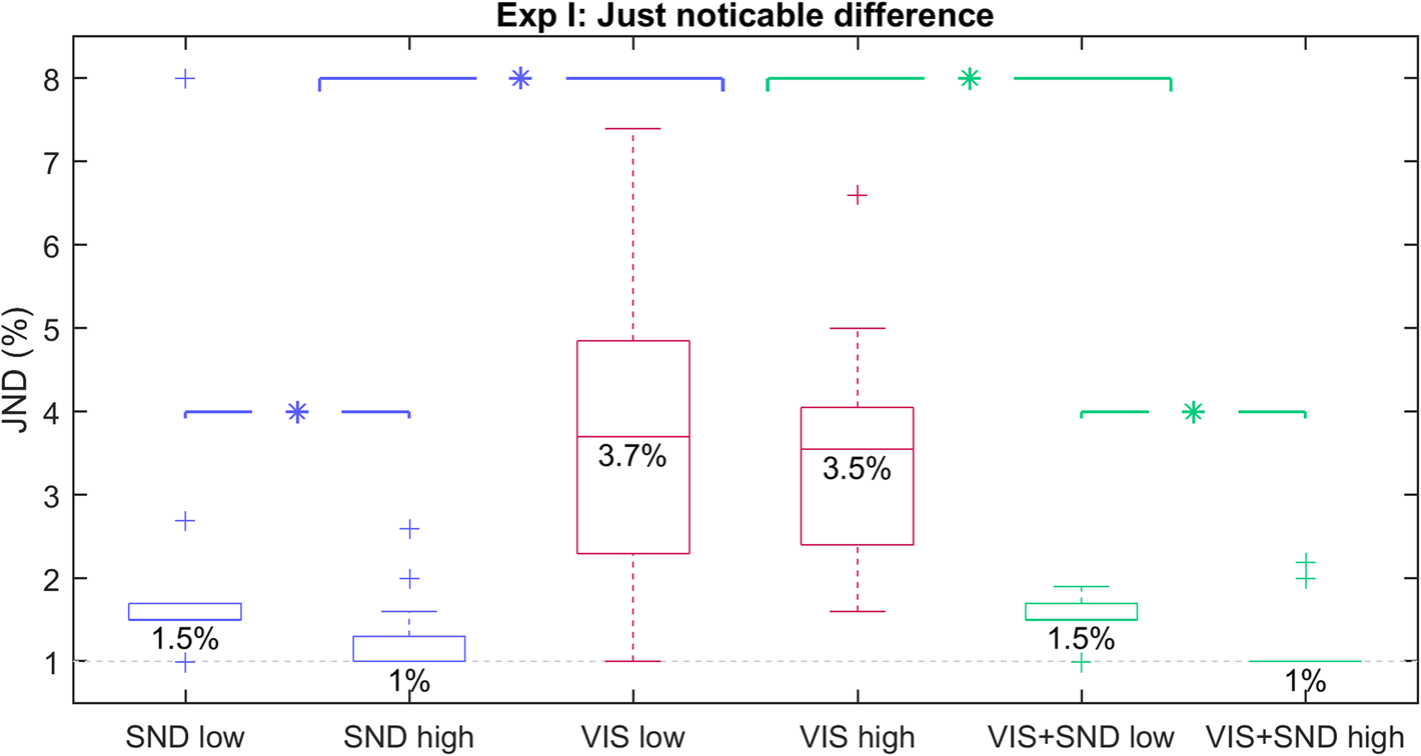
Overall JNDs for Exp I. The boxplots represent the overall JNDs across subjects for each feedback condition (*SND* in blue, *VIS* in red, *VIS + SND* in green) at the low (left) and high (right) standard-stimulus level, with the horizontal line indicating the median JND (also given as number), the box the interquartile range, the whiskers the range, and the pluses the outliers. Statistically significant differences between feedback conditions or between low and high levels within one feedback condition are depicted by asterisks

A representative example of a staircase trial is shown in Fig. 2. In general, at the high level of the standard stimulus, the subjects were able to discriminate the smallest possible change in prosthesis closing velocity (1%) in *SND* and *VIS + SND* conditions. The staircase sequence “saturated” at the difference of 1% in 9 and 10 out of the 12 subjects, respectively. However, this did not happen for any of the subjects in the *VIS* condition, and it was also uncommon at the low level of the standard stimulus regardless of the feedback condition (i.e., only a single subject per condition succeeded in saturating the staircase). Nevertheless, all feedback conditions resulted in a small JND with a median value of less than 4%. The summary results are shown in Fig. 3. The visual feedback was characterized with slightly higher JNDs. With median JND of 3.7 and 3.5% for the low and high level, respectively, *VIS* led to low but still significantly higher JNDs compared to *SND* (1.5 and 1%, respectively; *p* < 0.001) and *VIS + SND* (1.5 and 1%, respectively; *p* = 0.007). The median JND in the latter two conditions was very close or even equal to the smallest possible change in velocity (1%). The JND was slightly but significantly smaller at the high compared to the low standard-stimulus level for *SND* (*p* = 0.006) and *VIS + SND* (*p* = 0.004), while no significant difference was observed for *VIS*. Generally, these data show a surprisingly good discrimination no matter which type of implicit feedback was provided to the subjects.

### Experiment II: estimation task

During reinforcement learning, the success rates (median [interquartile range]) were 92% [8%] in *SND*, 88% [82%] in *VIS*, and 74% [18%] in *VIB*. Hence, the performance in *VIB* was worse compared to the two other conditions.

**Fig. 4:**
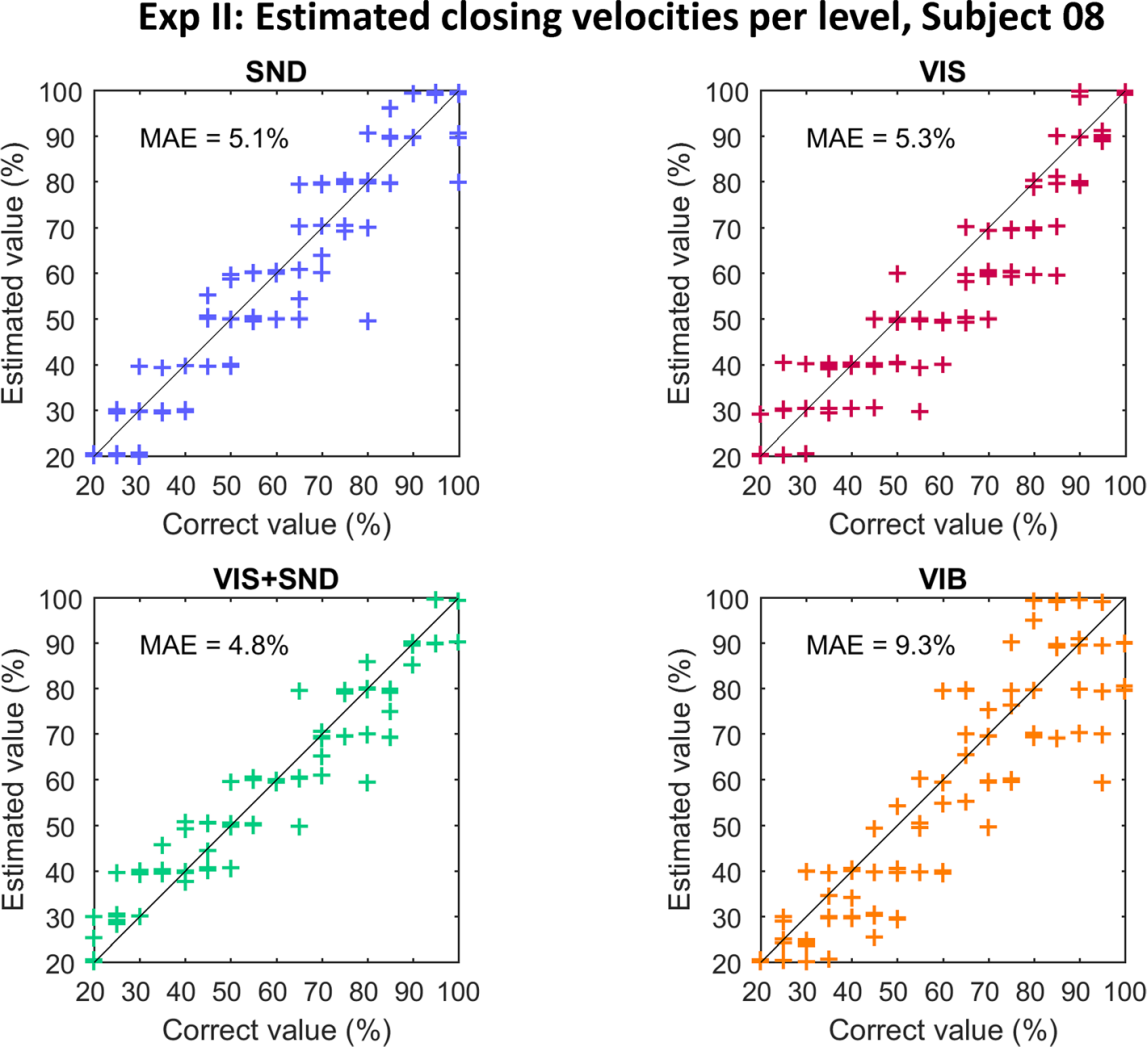
The results from the testing phase of one representative subject in Exp II. The estimated closing velocity (*VIS*, *SND*, *VIS + SND*) and vibration intensity (VIB) plotted against the correct stimulus value for Subject 8. The black line is the reference that would indicate the perfect performance (all responses correct)

The data from the testing phase of one representative subject are shown in Fig. 4, where the estimated versus correct closing velocities (*SND, VIS, VIS + SND*) and vibration levels (*VIB*) are plotted against each other for all feedback conditions and tested levels. The points are scattered around the optimal-performance line (black line), with a slightly worse quality of estimation in *VIB*.

**Fig. 5:**
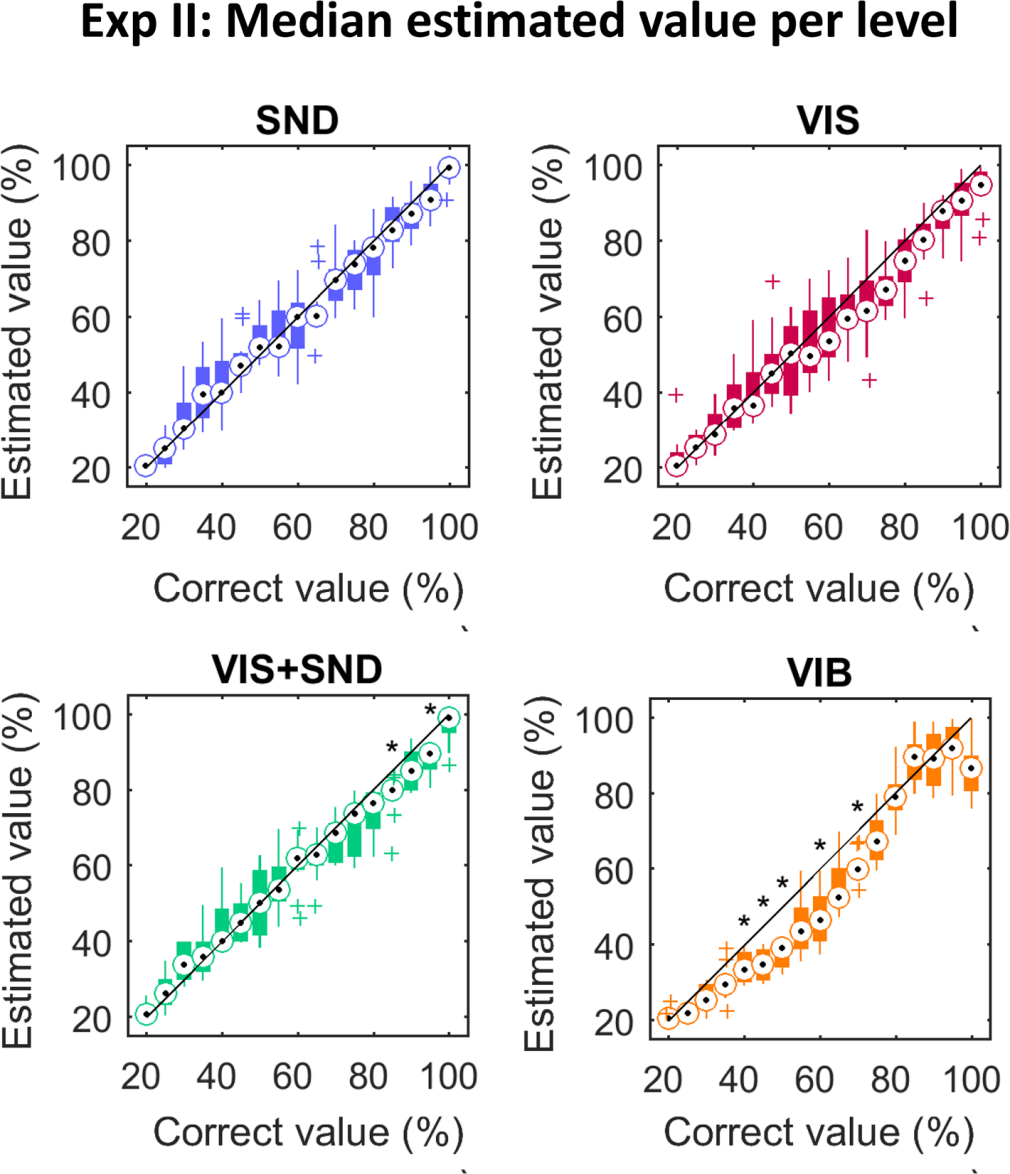
Median estimation per level for Exp II. Estimated value, i.e. estimated closing velocity/vibration, plotted against correct stimulus, i.e. generated velocity/vibration, with every boxplot representing the median (circle), interquartile range (box), range (line), and outliers (pluses) of all subjects’ medians for one level. Each subpanel represents one of the feedback conditions (see title). The black line is the reference line, which would reflect perfect responses. Significant differences (*p* < 0.05) from that line are indicated by asterisks

**Fig. 6:**
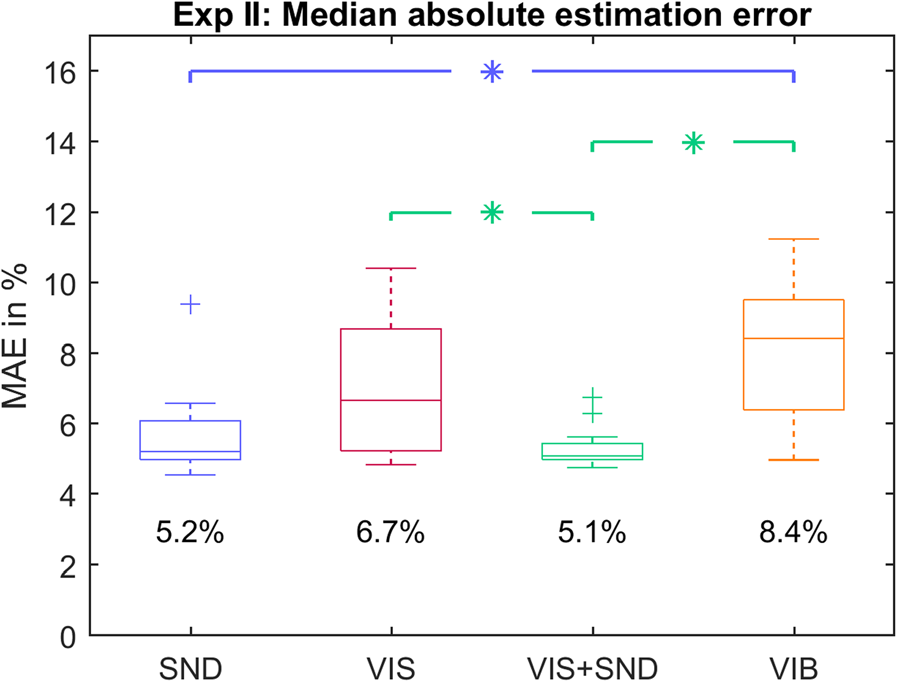
Overall median absolute estimation (MAE) error for Exp II. The MAE between estimated stimulus and correct stimulus with every boxplot representing the median (horizontal line), interquartile range (box), range (dashed line), and outliers (pluses) of all subjects’ MAE (obtained after pooling the absolute errors over all respective trials). Significant differences (*p* < 0.05) between feedback conditions are indicated by asterisks

The overall results are shown in Figs. 5 and 6. The medians of the estimated values followed consistenly the optimal-performance line (in black) across levels (Fig. 5). Statistically significant deviations from the line were not observed at any level for *SND* and *VIS*, at only two levels for *VIS + SND*, and at 5 out of 15 levels for *VIB*, indicating a general trend to underestimate the levels between 40 and 70% in this condition. Figure 6 shows the boxplots of the median absolute errors (MAE) across subjects (obtained by pooling trials across tested levels) for all feedback conditions. The performance was similar in *SND* (5.2% MAE) and *VIS + SND* (5.1% MAE). The estimation error in *VIS + SND* was significantly smaller than in VIS (6.7%; *p* = 0.005) and *VIB* (8.4%; *p* < 0.001) and the error in *SND* was significantly smaller than in *VIB* (*p* = 0.002). No significant difference in the quality of estimation was found between *VIS* and *VIB*.

**Fig. 7:**
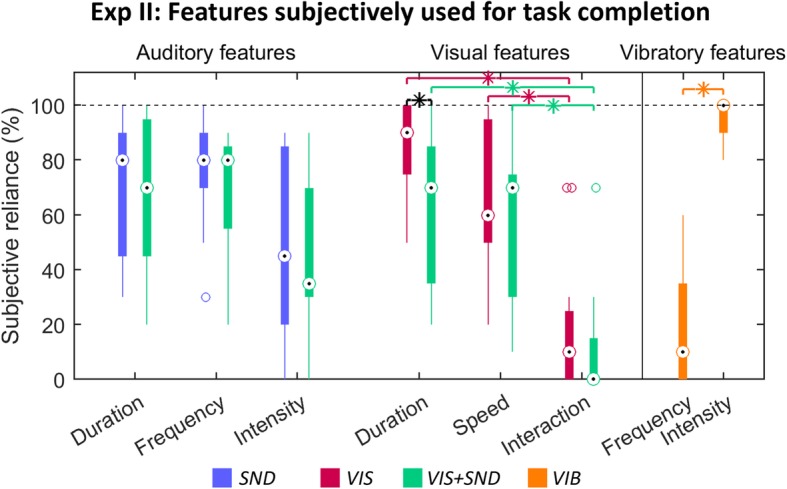
Subjective feature evaluation for Exp II. Boxplots reflecting the subjectively estimated reliance on the different available feedback sources for task completion in the four feedback conditions (colour-coded with blue for *SND*, red for *VIS*, green for *VIS + SND*, and orange for *VIB)*. Possible feedback sources for closing-velocity estimation were the duration of a stimulus (visual and/or auditory), the speed (visual), frequency (auditory), and intensity (auditory) of the closing hand, and the interaction between the fingers of the prosthesis and the wooden block (visual), while for vibration estimation the frequency and intensity of the tactor could be used. Every boxplot represents the median (circle), interquartile range (box), range (line), and outliers (pluses) of all subjects’ responses. Significant differences within feedback conditions are shown by asterisks in the respective color, black asterisks show differences between single-modality and full feedback tasks

**Fig. 8:**
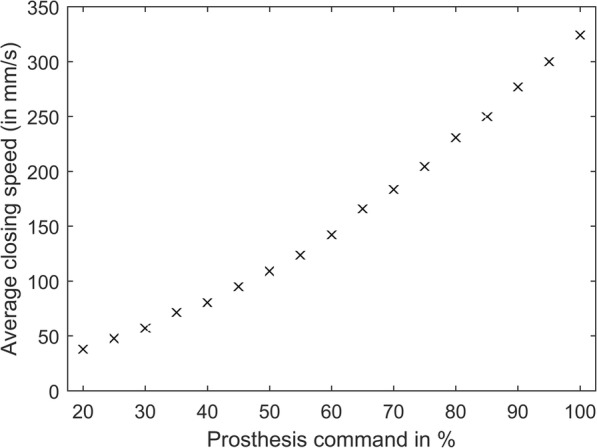
Pilot experiment assessing the closing speed for prosthesis commands

Figure 7 summarizes the results of the questionnaire in which the subjects reported on the relevance assigned to different features of the feedback sources. Regarding the auditory features, most subjects seemed to focus on closing duration (median 80% for *SND* and 70% for *VIS + SND*) and frequency (80% for *SND* and 80% for *VIS + SND*) instead of intensity (45% for *SND* and 35% for *VIS + SND*). However, this trend was not significant neither in *SND* nor in *VIS + SND* likely due to high variability across subjects. Regarding visual features in *VIS* (Friedman test *p* < 0.001) and *VIS + SND* (Friedman test *p* < 0.001)*,* the *s*ubjects were significantly more attentive to the duration (90% for *VIS, p* < 0.001*,* and 70% for *VIS + SND, p =* 0.001) and closing speed (60% for *VIS, p =* 0.001*,* and 70% for *VIS + SND, p* < 0.001) than to the interaction between prosthesis and wooden block (10% for *VIS* and 0% for *VIS + SND*), which was hardly exploited at all. No significant differences in used features between full (*VIS + SND*) and partial (*SND* or *VIS*) feedback were observed, except for a higher focus on the closing duration in *VIS* compared to *VIS + SND* (shown by a black asterisk in Fig. 7; *p* = 0.002)*.* In *VIS + SND*, the subjects concentrated similarly on visual and auditory features, with none of the two modalities being rated significantly higher than the other. For the vibratory task, the subjects clearly focused significantly more on the intensity (median 100%) than on the frequency information (10%; *p* < 0.001). Concerning the subjective estimation of performance, they reported a subjective MAE of 10% (IQR 6%) for *SND*, 17% (IQR 8%) for *VIS*, 9% (IQR 7%) for *VIS + SND*, and 15% (IQR 12%) for *VIB*.

## Discussion

The present study investigated the psychometric properties of the incidental feedback in prosthetic grasping. The quality of feedback sources inherent to a prosthetic device (vision and sound) was assessed by conducting psychometric tests and it was compared to the quality of feedback provided by a vibration motor, which is a common solution to transmit somatosensory feedback in prosthetics. The experimental results demonstrated that the visual and auditory feedback of the prosthesis motion could be used to estimate the closing speed with high reliability. In addition, the estimation using sound and vision alone or in combination was significantly better compared to the vibration feedback.

With sound and vision combined or only sound alone, the obtained JND was below 2% at both high and low closing speeds. With vision alone, the JND was somewhat higher (below 4%), which is in agreement with who reported that auditory feedback is more useful than visual feedback for temporal discrimination. The present study therefore demonstrates that the inherent resolution of the incidental feedback sources is truly remarkable. Only by relying on the incidental feedback, the subjects were capable of discriminating changes in the prosthesis closing velocity that are close to the minimum increment/decrement in velocity that can be commanded to the prosthesis (1%). In addition to detecting a change in velocity, the subjects could estimate the absolute value of the closing speed with high precision. When estimating a closing speed randomly selected from the full working range, the average error was around 5% for *SND* and *VIS + SND* and around 7% for *VIS*. As explained in Introduction, the estimation of prosthesis closing velocity can be used to control grasping force [48]. The present study shows that the incidental feedback can provide such an estimate rather precisely, and this explains why the incidental sources can be so useful for prosthesis control [46].

The combined visual and auditory feedback resulted in the best performance, as indicated by both lowest MAE and low variability across subjects. This demonstrates that the subjects could fuse the incidental sources of feedback to improve the estimation of the prosthesis state. The studies of human motor control have shown that human subjects can integrate sensory information from multiple sources based on their relative uncertainty [51–53].

The present study demonstrated that the inherent prosthesis feedback could provide reliable closing-speed information to the prosthesis user after only a brief training (< 5 min). When the same information was transmitted using only vibration feedback, the quality of estimation was significantly worse (MAE ~ 8%). Importantly, the results for *VIB* condition might be different if another coding scheme would be used. We have used a linear mapping since this is a simple and therefore a common choice in the literature [36, 54, 55]. In reality, the mapping from the intensity of stimulation to the perceived intensity follows a power function [17, 56]. And indeed, such a trend can be seen in Fig. 5 (*VIB*), where the subjects underestimated consistently in the middle range of intensities. This effect, although significant, was not substantial, possibly because we have modulated the frequency in addition to the intensity to further facilitate the discrimination. Nevertheless, better estimation (e.g., less undershooting) in the *VIB* condition might be obtained if a different transfer function would be used; this remains to be tested.

The above results might indicate that adding vibration as a supplemental feedback to communicate the variables that can be already estimated from the intrinsic sources directly (closing velocity) or indirectly (grasping force) could have limited impact on performance of prosthesis control. The intrinsic feedback seems to be sufficient so that there is not much margin for improvement. Of course, this assumes that the subject can indeed observe the prosthesis while closing. Also, the performance of *VIB* was worse already in the learning phase, which was rather short in the present study. It would be interesting to investigate the trends as well as the final performance in each feedback modality after a prolonged learning.

There was a substantial variability in the subjective reports about the relevance of different features of the feedback sources. Nevertheless, in all the incidental feedback conditions, the subjects focused on the temporal aspects of the feedback stimulus, namely, the stimulus duration. On the contrary, the cues from the mechanical interaction between the prosthesis and the object seem not to be exploited. Finally, although the sound frequency was appreciated by the subjects in the acoustic feedback condition, when using vibration feedback, they focused substantially more on the intensity. This could be due to the frequency range of the C2 tactor, which does not include low frequencies (< 30 Hz), whereas the intensity was modulated between 8 and 71%. Interestingly, when estimating their own performance, the subjects ranked the conditions according to the objectively measured results (i.e., *SND* and *VIS + SND* performed better than *VIS* and *VIB)*. However*,* they reported a higher subjective uncertainty, i.e., the perceived estimation error was greater than experimentally observed.

The present study is an important initial step towards understanding the interaction between incidental and explicit feedback in closed-loop prosthetics. In another prosthesis, the vision and sound feedback would be different in quality and/or quantity (e.g., sound pitch and loudness), but the main principle would still hold (e.g., the movement speed and therefore sound associated to force).

## Limitations

In real life scenarios, the usefulness of incidental (visual and auditory) feedback can decrease in the presence of overlaying and distracting sensory inputs, e.g. in noisy environments or when the visual attention is diverted from the prosthesis. Also, wearing a cosmetic glove over the prosthesis, which is usual in clinical applications, might decrease the loudness and discriminability of the auditory information. In this case, supplemental vibro- or electrotactile feedback might be beneficial since it is transmitted through a different sensory channel (sense of touch).

The experimental task in the present study resembled routine grasping, in which the prosthesis closes at a constant velocity to produce proportional force upon contact. However, modulating the force after the prosthesis has closed around an object is a different task, in which supplemental force feedback, delivered using vibration or other methods, is likely to be useful. In this case, different sources of feedback might be relevant. For example, if the prosthesis holds a compliant object while closing, the subjects could estimate the grasping force from the amount of object deformation.

In the present experiment, the computer sent a command to the prosthesis to close at a constant velocity. In real-life application, the prosthesis will be controlled by a user generating myoelectric commands. The myoelectric signals are known to be variable and this is likely to cause variability in the prosthesis closing speed (even if the user would like to produce a constant velocity). This can challenge the estimation of prosthesis variables from the incidental sources. Nevertheless, recent studies show that incidental feedback can be useful despite this uncertainty [46, 48]. However, it would be useful to measure the psychometric parameters in this context, namely, during realistic proportional control and feedback. In this case, a proprioceptive information from the sense of muscle contraction could be used additionally to vision and audition [46]. Finally, the results reported here were observed in naïve able-bodied subjects; the implications obtained, however, should be even more relevant for experienced prosthesis users, who had enough time and practice to learn to interpret the prosthesis sound and movement.

## Conclusion

The present study shows that the implicit feedback coming from a hand prosthesis can be an important source of information when estimating the prosthesis state. The subjects were able to accurately discriminate (experiment I) and estimate (experiment II) the velocity of prosthesis closing using vision and sound, and they could even fuse the two incidental feedback sources to improve the estimation quality. This is a strong indication that the feedback, which is intrinsically available from the prosthesis, can be usefully exploited for the control of prosthesis grasping. This point should be considered when designing supplemental feedback provided through sensory substitution (e.g., electro and vibrotactile stimulation). If supplemental feedback is to be efficient, it needs to be of a higher quality or should transmit information that is not already available implicitly (e.g., EMG feedback [41, 57, 58]).

## Data Availability

The datasets used and/or analysed during the current study are available from the corresponding author on reasonable request.

## Appendix

To measure the prosthesis closing speed, the hand was mounted on a vice and fully opened in palmar grasp. A Matlab program was implemented that repeatedly closed and opened the prosthesis through a full range of speeds. The normalized prosthesis commands in the range between 20 and 100%, in steps of 5%, were repeated three times and the time needed for closure from the fully open prosthesis (12 cm aperture according to the manufacturer) to the fully closed prosthesis was measured. Then, the average closing speed was computed by dividing the aperture (12 cm) by the mean closing time per prosthesis command. The profile of obtained closing speeds is depicted in Fig. 8. It can be seen that the two standard speeds used for JND measurements correspond to the two segments of the profile with different slopes.

## Abbreviations

CV: Comparison value
Exp I: Experiment I
Exp II: Experiment II
JND: Just noticeable difference
MAE: Median absolute error
*SND*: Acoustic feedback
SV: Standard value
*VIB*: Vibrotactile feedback
*VIS + SND*: Combined feedback
*VIS*: Visual feedback
